# Editorial: 21st century advances in type 1 diabetes research and immunotherapy

**DOI:** 10.3389/fimmu.2023.1213417

**Published:** 2023-05-12

**Authors:** David Bleich

**Affiliations:** Division of Endocrinology, Diabetes, & Metabolism, Rutgers New Jersey Medical School, Newark, NJ, United States

**Keywords:** type 1 diabetes, T cells, immunology, microbiome, neoantigen, GAD 65, LADA (latent autoimmune diabetes in adults)

The immunology of human type 1 diabetes (T1D) is complex and lacks detailed understanding. Rodent models of disease recapitulate certain molecular and genetic aspects of human disease but have not yielded high quality molecular targets to prevent or reverse progressive pancreatic beta-cell destruction. The predominant paradigm for the past 30 years of hypothesis-driven research is that a handful of carefully identified auto-antigens (both beta-cell specific and non-specific) might be able to induce tolerance in individuals at high risk for developing T1D (typically first-degree relatives of T1D subjects who have multiple T1D autoantibodies). Tolerance induction has been used successfully for decades in allergy immunotherapy, but so far has not been translated over to T1D. Such studies include major immunotherapy trials with insulin, insulin peptides, and glutamic acid decarboxylase 65 (GAD; a major T1D autoantigen). New research suggests that immune-mediated T1D might not follow a single molecular roadmap but develops through a variety of derangements culminating in beta-cell destruction. Moreover, novel neoantigens have been discovered with enhanced T-cell reactivity that might prove to be better for inducing tolerance. While antigen-specific immunotherapy for T1D has yet to demonstrate clinical success, autoantibodies continue to be useful tools for identifying at risk individuals and T1D subtypes.

In this special issue of Frontiers, Peng et al. define three subtypes of diabetes in their large multi-institutional Chinese cohort: latent autoimmune diabetes in youth (LADY; age between ≥15 to <30 years, no ketoacidosis and insulin independence for 6 months, disease duration < 1 year, and autoantibody positive with either GAD, IA-2A, or ZnT8A), latent autoimmune diabetes in adults (LADA; age >30 years, no ketoacidosis and insulin independence for 6 months, disease duration <1 year, and autoantibody positive with either GAD, IA-2A, or ZnT8A) and type 2 diabetes (>30 years, disease duration <1 year, and autoantibody negative). Using epitope specific GAD assays that identified C-terminal, Middle region, and N-terminal autoantibodies, they determined that young T1D (<15 years), LADY, and old T1D (≥15 and <30 years) subjects had higher reactivity to multiple GAD epitopes than LADA patients. Moreover, T1D and LADY subjects had more high-risk susceptibility alleles for disease than LADA subjects. The authors suggest that different immune activity exists between T1D and LADA subjects.

LADA has been defined primarily in Caucasian populations, but less so in Asian populations. With a population of ~ 1.4 billion people, China has a growing number of individuals with diabetes. Qiu et al. performed a systematic review of epidemiology, clinical characteristics, genetics, and immunology of LADA in this growing Chinese population. Importantly, they note that high risk allele HLA DR9/DR9 in Chinese LADA contrasts the well-established DR3/DR4 in Caucasian populations. In addition, GAD antibody positivity was only ~75% in Chinese LADA subjects compared to 90-95% in Caucasian disease. Additional features of LADA in China are well described including clinical manifestations and treatment options.

Pathogenic effector T-cells (both CD4^+^ and CD8^+^) infiltrate the pancreatic islets in T1D. Identification of autoantigens that stimulate pathologic T cells has become a highly valued research endeavor. Li et al. used NOD mice as a T1D model to demonstrate the utility of a novel monoclonal antibody that targets the highly diabetogenic islet amyloid polypeptide (IAPP) K20. The K20 peptide is formed from IAPP that undergoes post-translational modification through a disulfide bridge between two cysteine residues and thereby becomes an autoantigen for the diabetic T cell clone BDC 5.2.9. LD96.24 is a monoclonal antibody that recognizes the IA^g7^-K20 disulfide loop thereby blocking its interaction with the BDC T cell receptor. When prediabetic and newly diabetic NOD mice were treated with this monoclonal antibody disease prevention was sustained for the treatment duration.

The B-chain of insulin (amino acid sequence B:9-23) has been considered a predominant autoantigen in T1D. Wenzlau et al. present exciting new data about a post translational modified (PTM) hybrid insulin peptide (HIP) called HIP6.3 that results from the fusion of insulin B:9-23 with a ProSAAS sequence (ProSASS is an endogenous inhibitor of prohormone convertase). When used in a stimulation assay with the highly diabetogenic T cell clone BDC-6.9, this neoantigen generated a 4 to 5-fold increased interferon-gamma response. This neoantigens generates more inflammation and immunity in T1D than conventional antigens.

Under appreciated is the role of lymphotoxins (LTs) in immune inflammatory disease. Liu et al. provide a comprehensive overview of LTs and their indirect role in cytotoxicity towards pancreatic beta cells. LTs mediate formation of tertiary lymphoid organs that are seen in NOD mice and perhaps play a role in T cell recruitment to the pancreatic islets.

In addition, Yang et al. present an elegant and comprehensive review of post-translational modification (PTM) of proteins in autoimmunity and beta cell metabolism. This detailed publication provides a fundamental roadmap for understanding metabolic changes that create neo-antigens, alter T-cell metabolism, and improve biomarker sensitivity in T1D and autoimmune conditions. Highlights include detailed explanations of peptide citrullination, T-cell transition from oxidative phosphorylation to glycolytic metabolism, and PTM of novel T1D biomarkers. This is a must read.

Turning to clinical correlates between effector T cells and T1D progression, T cell metabolism was assessed in T1D subjects over 36 months. Complex interactions occur between cellular metabolism of glucose or fatty acid and T cell function. Simplistically stated, effector T cells have increased autoreactivity when their metabolic program uses glucose rather than fatty acids. Tang et al. evaluated glucose and fatty acid metabolism in CD4^+^ and CD8^+^ T cells from 86 Chinese T1D subjects and 45 non-diabetic controls and followed them for 3 years. T1D subjects with low T cell glucose uptake at entry maintained higher c-peptide levels and beta cell function compared to those individuals with high glucose uptake. Therefore, T-cell metabolism now appears to be an important component of sustained autoimmunity in T1D.

Last, the bacterial flora in the gut plays an important role in T1D susceptibility through host-microbe interactions. Studies in monozygotic twins show discordance in T1D development which suggests that non-genomic factors play a role in the disease. It is proposed that altered microbiome in the gut leads to proinflammatory responses through numerous mechanisms including direct signaling from bacterial metabolic products to receptors that regulate immune responses in the human host. Therefore, one twin might have a different microbiome than the other twin and perhaps, a different immune system response. Host-microbiota interactions in T1D is extensively reviewed by Majumdar et al. as a capstone for this special edition.

## Author contributions

The author confirms being the sole contributor of this work and has approved it for publication.

